# Operationalizing AI ethics in medicine—a co-creation workshop study

**DOI:** 10.1186/s12910-025-01317-y

**Published:** 2025-10-29

**Authors:** Luis M. Lopez-Ramos, Gabriele Pluktaite, Cathrine K. T. Bui, Julia Amann, Tamarinde Haven, Vince I. Madai, The VALIDATE Consortium

**Affiliations:** 1 Department of Holistic Systems, Simula Metropolitan Center for Digital Engineering, Oslo, Norway; 2 QUEST Center for Responsible Research, Berlin Institute of Health, Charité – Universitätsmedizin Berlin, Berlin, Germany; 3 Strategy & Innovation, Careum Foundation, Zurich, Switzerland; 4 Department of Methods and Statistics, School of Social and Behavioural Sciences, Tilburg University, Tilburg, The Netherlands

**Keywords:** AI ethics, Co-creation, Ethical principles, Content analysis

## Abstract

**Background:**

A majority of AI ethics frameworks focus on high-level principles but lack actionable guidance. Effectively implementing AI in projects requires the operationalization of AI ethics, translating principles into requirements. This paper proposes a novel method for operationalizing AI ethics through co-creation workshops.

**Methods:**

The study adopted a qualitative, participatory research approach. Stakeholders from the VALIDATE project, a Horizon Europe action developing an AI-based clinical decision support system for stroke patient stratification, were divided into five diverse teams. The workshops aimed to: (i) determine the low-level ethical requirements stakeholders considered essential for the project, and (ii) identify requirements not included in the EU Trustworthy AI Guidelines. The methodology included storytelling, content analysis to identify ethical issues and dilemmas, eliciting quantifiable requirements using a standardized planning language (Planguage), and a procedural feedback process.

**Results:**

The workshops identified *explainability, privacy, model robustness, model validity, epistemic authority, fairness,* and *transparency* as key ethical issues. Participants drafted low-level requirements related to *privacy*, *explainability*, *transparency*, and *validity*. Six issues (*time sensitivity, validity, prevention of harm to patients, patient-inclusive care, quality of life,* and *lawsuit prevention)* could not be mapped to the EU Guidelines. Participants did not draft requirements in relation to the latter issues. Challenges included the diverse interpretations of concepts, such as *validity*. Participants generally had favorable impressions of the workshops, although they found formulating requirements in Planguage format more challenging than storytelling and topic prioritization.

**Conclusions:**

The workshops elicited concrete, quantifiable, and actionable requirements, which were useful in developing a project-specific ethical framework. The proposed methodology is resource-efficient and requires fewer AI ethics experts than existing methods while remaining compatible with established guidelines. Procedural feedback results suggest that participants and facilitators would benefit from additional training in the use of Planguage. Potential challenges included the impact of power dynamics among participants on discussions, blind spots due to overlooked issues, and the absence of stroke patients among the participants.

**Supplementary Information:**

The online version contains supplementary material available at 10.1186/s12910-025-01317-y.

## Background

The surge of digitally accessible medical data has significantly increased the appeal of artificial intelligence (AI) in healthcare. AI tools offer novel and promising possibilities for enhancing healthcare efficiency and effectiveness [1], especially in the form of clinical decision support systems (CDSS). Such AI systems can address serious challenges in contemporary medicine, such as rising costs associated with medications, early disease detection and management, and the need for personalized treatment plans for complex conditions such as cancer [2–5]. Early prognosis of the evolution of an acute disease can be beneficial for treatment decision making in time-constrained situations.

However, harnessing AI's full potential in healthcare also hinges on establishing relevant ethical development processes. At first glance, abundant resources appear to be available for achieving ethical AI development. Already by mid-2019, there were over 80 publicly available ethics guidelines globally, emphasizing key principles such as transparency, justice and fairness, non-maleficence, responsibility, and privacy [6]. The Ethics Guidelines for Trustworthy AI, published by the European Commission’s High-Level Expert Group (HLEG) on AI, define seven key requirements: Human agency and oversight; Technical robustness and safety; Privacy and data governance; Transparency; Diversity, non-discrimination, and fairness; Societal and environmental well-being; and Accountability. These requirements serve as foundational principles that AI systems should adhere to in order to be deemed trustworthy [7].

Despite an extensive body of guidance, however, the AI landscape is riddled with AI applications [8], including in healthcare, whose use is challenged by ethical issues, e.g., racial bias and exacerbation of socioeconomic inequities [9], lack of equity in COVID-vaccine allocation [10], and cybersecurity breaches affecting the clearing of medical claims [11]. A key issue arises from the abstract nature of most ethical frameworks: they focus on high-level principles rather than actionable guidance [6, 12, 13]. The principlism approach pioneered by Beauchamp and Childress focuses on abstract moral principles rather than detailed rules or operational guidance [14]. This feature may have influenced the structure of subsequent AI ethics frameworks, although this connection would warrant further analysis.

To date, most AI ethics tools reviewed by Morley et al. [12] stop at the level of principles and do not translate into practical guidance. Many frameworks, as mapped by Jobin et al., [6] reiterate similar high-level principles with little actionable detail. Mittelstadt [13] further argues that principles alone are insufficient to ensure that AI is developed and deployed ethically.

Empirically, most available frameworks offer little practical insight and hence fail to address implementation [15]. In other words, the current ethical landscape in AI predominantly highlights *what* needs to be done without addressing *how* to do it in practice [12]. Such vague directives fail to address relevant normative challenges arising in development [13]. Consequently, there is a significant gap between ethical principles and their translation into actual practices [16].

The process of translating high-level principles into actionable, practical guidance is referred to as *AI ethics operationalization* [12]. A meta-analysis reviewed over 100 articles on ethics operationalization, identifying a wide range of technical approaches [17]. Some of the important utilized approaches include the bottom-up methodology of embedded ethics [18], which integrates ethics through stakeholder collaboration, or Value Sensitive Design [19], an iterative methodology involving conceptual, empirical, and technical investigations to integrate ethical values throughout the design process [20]. In recent years, the Z-inspection process has emerged as a holistic method of assessing and improving the ethical issues of existing AI tools, particularly in healthcare. This framework engages a multidisciplinary team in analyzing various aspects of AI through ethical considerations and dilemmas, with the European Union (EU) Trustworthy AI guidelines [7] at the forefront. However, while these approaches can be comprehensive and effective, they are resource-heavy and difficult to scale amidst the growing ubiquity of AI technologies.

Thus, alternative, resource-efficient, and scalable approaches are needed to support the operationalization of AI ethics. This paper proposes and explores a novel method centered on the use of co-creation workshops to support the translation of high-level ethical principles or guidelines into actionable requirements. In co-creation, relevant stakeholders work together to generate value and meaning [21]. Recently, a study discerned inclusive co-creation as one of the key drivers of digital health innovation, which can prevent harmful ideas at the design stage, address overlooked patient problems, and prioritize stakeholder concerns [22]. Workshops, in general, have proven effective in fostering consensus, idea generation, and negotiation among stakeholders while also mitigating conflicts and enhancing mutual understanding [23]. A recent example focused on accountability and risk management in AI, gathering insights through workshops and pinpointing the essential features for risk management and areas needing more clarity [24]. Another study utilized workshops of international experts to explore ethical, legal, and social issues related to social robots, compiling the findings to discern general trends and urgent concerns [25]. Despite these initiatives, to our knowledge, co-creation workshops have not been utilized beyond addressing particular ethical issues.

In the present study, we organized a series of virtual co-creation workshops with stakeholders within the VALIDATE project, a Horizon Europe research and innovation action (RIA) with the goal of developing a CDSS for stroke patient stratification [26, 27]. Three co-authors (CB, LLR, VM) acted as facilitators, and project staff across all disciplines represented in the VALIDATE project were involved as workshop participants (Here, “participants” refers specifically to the subset of VALIDATE project staff who took part in the study workshops, distinct from the broader group of individuals employed in the project.). The workshops involved storytelling to identify ethical issues, their ranking, and their preliminary formalization as quantifiable requirements, which the facilitators then refined as part of the ethical framework. A procedural feedback process, including participant questionnaires, was employed. Content analysis was applied to the workshop transcripts to extract priorities and connections among the identified themes.

### Research questions

The workshops were designed to answer the following research questions (RQs): *RQ1. What do VALIDATE staff members consider to be key low-level requirements for the use of AI in stroke? RQ2. Can the use of co-creation workshops lead to the identification of requirements that cannot be mapped to the EU Trustworthy AI Guidelines?* The rationale behind RQ1 was to gather participant input to guide the practical development of a trustworthy AI system by setting priorities and concrete goals that could benefit similar projects. We use the term low-level requirements to refer to concrete, operational specifications that refine high- and mid-level ethical principles into actionable terms specific enough to inform system design or evaluation. This aligns with the general distinction made in requirements engineering between high-level business or stakeholder needs and detailed functional and non-functional solution requirements [28]. The rationale behind RQ2 was to evaluate whether the co-creation process could uncover additional ethical considerations beyond standard trustworthy AI concepts.

## Materials and methods

### Data collection

#### Structured group storytelling

Participants were prompted to share stories that highlight ethical dilemmas or challenges, with preference given to real-life experiences, though hypothetical scenarios were also accepted. While stories related to stroke care were prioritized, ethical issues from other contexts were also deemed relevant. Using narratives promotes moral reasoning and ethical decision-making [29], and allows these experiences to be abstracted into needs, goals, and constraints. This method does not require specific training from participants [30] or facilitators [31], and fosters active engagement, listening to peers' stories enhances memory retention and idea expression.

In our study, storytelling was used within a co-creation workshop format rather than a traditional focus group. While focus groups typically aim to elicit diverse views through discussion, co-creation workshops prioritize collaborative problem-solving and shared ownership of outcomes. Integrating storytelling into this format enabled participants to elicit experiential knowledge in a structured yet creative environment, allowing them not only to surface ethical concerns but also to frame them as concrete requirements.

A detailed description of the storytelling approach is available in the online protocol [32], and the full set of discussion prompts is provided in Table S2 of the Appendix.

#### Planguage

Quantified metrics enhance transparency and accountability, providing a clearer measure of progress compared to simpler approaches such as binary checklists. A suitable tool for this purpose is a planning language (specifically, Planguage [33]), an interactive tool used to quantify outcomes from processes or tasks. It has been selected for its quantifiability, structured measurability and traceability, rigor, transparency, and accessibility to diverse stakeholders. In this study, Planguage was employed to address stakeholder values and viewpoints visually and explicitly [33].

In the context of information technology projects in healthcare, stakeholders often face difficulties in aligning on system analysis and expected results [34]. The Planguage format is a systematic way of expressing requirements in a measurable form, by defining parameters, scales, and degrees of completion. Whenever the degree of completion of a requirement is not sufficiently described by a binary (done/not done) scale, expressing it via Planguage allows enough resolution to evaluate the need for (re)work to improve the degree of completion if necessary.

For instance, in the following example, Planguage allows for explicit articulation of project goals:

All [development materials] are stored via [practices for privacy],

with practice enabled before [date].

Here, the brackets contain key parameters. "[Development materials]" might refer to data, models, predictions, or AI results. "[Practices for privacy]" could include encryption, password protection, or server security measures. The "[date]" parameter can refer to a specific project milestone or deadline.

#### Workshop setting

This study was conducted as part of the Horizon Europe project VALIDATE, which aims to develop an AI-based CDSS for stroke patient stratification [27] with a central focus on ethically grounded AI development. As the project is designated by the European Commission as a blueprint reference for future trustworthy AI projects in clinical settings, the workshop methodology aimed to explore the operationalization of AI ethics through co-creation with project stakeholders.

At the time when the workshops were conducted (months 8 and 9 of the project, which has a total duration of 48 months), project staff had started defining the technical and design requirements of a demonstrator, with the goal of advancing the AI system from technology readiness level (TRL) 3 to TRL 6 by the end of the project. The TRL scale is used to assess the maturity of a technology, especially in EU-funded research projects. TRL 3 corresponds to experimental proof of concept, where the underlying technological principles have been validated in a controlled setting. TRL 6, in contrast, refers to a technology demonstrated in a relevant (in this case, clinical) environment, with a functioning prototype tested under realistic conditions [35]. These activities included defining technical machine learning (ML) requirements, developing a clinical ML model, user app design, specification analysis, business model planning, and clinical validation planning for a retrospective study and a prospective observational study.

### Participants

Twenty participants from 8 institutions were included in the workshops. They were selected among staff from the VALIDATE project holding scientific, technical, or managerial roles, and were contacted via email. Exclusions were made for individuals unable to attend all sessions or those involved in the study design. Due to scheduling difficulties, five of the individuals who were originally contacted and had agreed to participate later withdrew, resulting in a final sample of 20 participants. The sample was split into 5 teams of 3–5 participants: given the time constraints, we considered that in a team with 6 or more participants, the risk of one or more participants not contributing significantly was too high. Teams were intentionally composed to maximize diversity, prioritizing that each team included at least one clinician, and aiming for a proportion as balanced as possible of AI researchers, software developers, and patient representatives. People from the same organization were assigned to different teams, when possible, to mitigate potential biases due to power dynamics. This interdisciplinary approach aimed to foster balanced discussions and mitigate biases. Participants were instructed to isolate themselves, i.e., find a place where others could not overhear them (e.g. meeting room) so they could share their stories and opinions freely and comfortably.

### Facilitators

Three facilitators (LLR, CB, and VM) with expertise in AI for medicine, AI ethics, and workshop facilitation conducted the workshops. They guided the participants through the planned activities, ensuring consistency and validity while minimizing the risk of priming. They refrained from sharing personal opinions or introducing specific ethical issues, allowing the issues and concerns to emerge naturally from participants' experiences. Facilitators were encouraged to keep a private reflexivity diary to increase the opportunity to identify their own biases and how their work influences the ethics awareness of the project staff and the workshop outcomes.

### Workshops methodology

The workshops were inspired by earlier efforts to co-create an AI ethics framework with project partners involved in the Horizon2020 Project, PRECISE4Q [36]. The workshops were designed to maximize the extraction of participant knowledge, experience, values, and perspectives in a short time frame (two 2-h sessions). A combination of co-creation, structured group storytelling, and Planguage technique was used, with content analysis applied to analyze the data. A co-creation approach engaged participants in decision-making, ensuring that all voices were considered equally, regardless of power dynamics [37]. This bottom-up process facilitated the integration of diverse perspectives into actionable outcomes [38]. The methodology was pilot-tested with a medical doctor and a software engineer, who provided feedback on the clarity and structure of the workshop. No repetition of the workshop sessions was necessary. A detailed description of the workshop and study methodology is available online in the protocol. (preregistration: [32]).

### Workshop structure

The workshops were conducted virtually to accommodate participants from various European countries, using Microsoft Teams [39] for videoconferencing. Facilitators used Mural [40] to create digital collaborative whiteboards where participants could freely brainstorm, contribute, and organize their ideas. SessionLab [41] was employed by facilitators to manage and structure the workshop agenda.

**Fig. 1:**
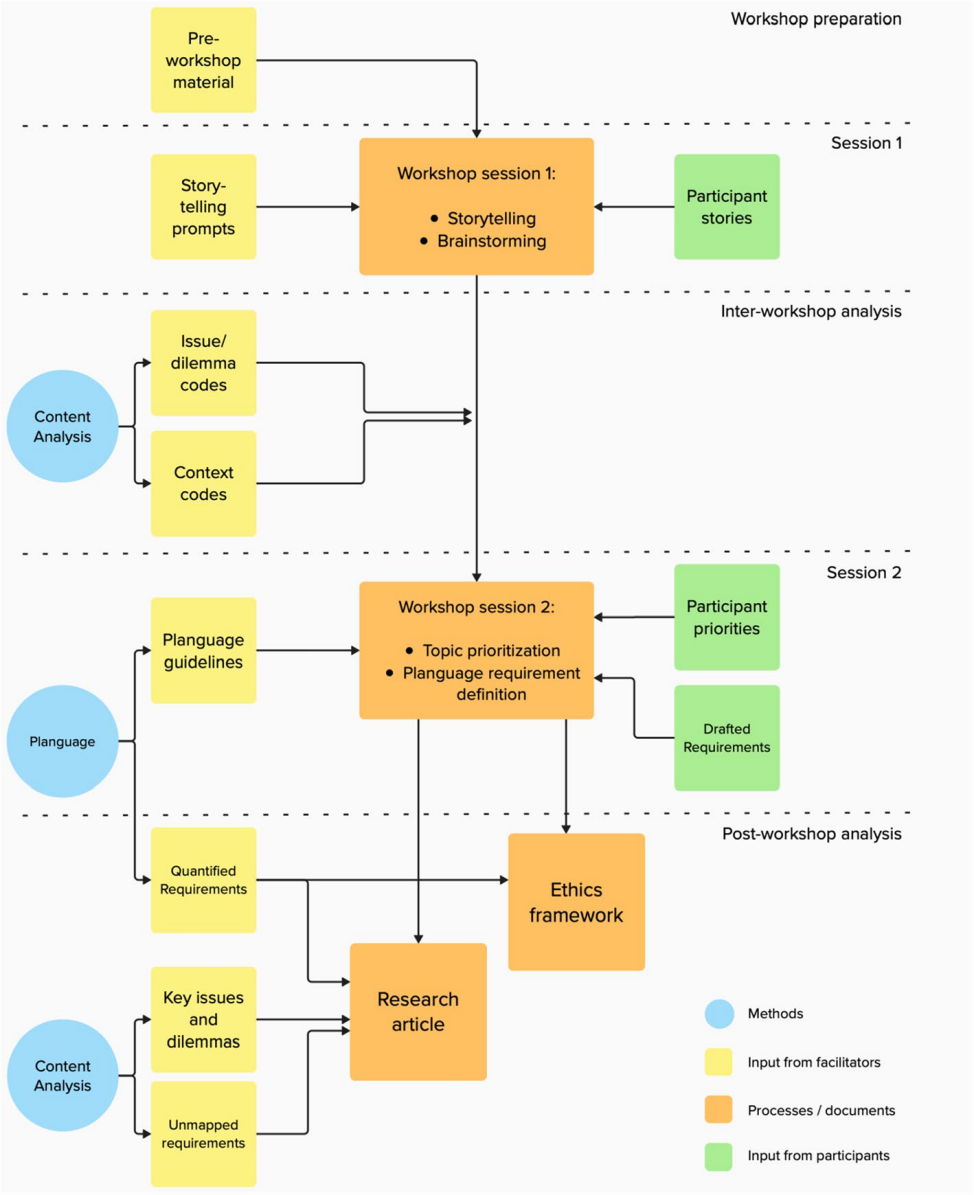
Flowchart showing the structure of each workshop thread. Created using Mural [40]

Each workshop was structured across five phases: preparation, first session, inter-session analysis, second session, and post-workshop analysis. The workshops were organized into five parallel threads, one per team, ensuring that all phases were executed within the allocated time frame. A flowchart of the workshop structure is shown in Fig. 1.

#### Preparation

The preparation phase involved two sets of materials: (a) pre-workshop handouts and (b) materials for use during the sessions. The pre-workshop package included an executive summary of the project, a description of the AI system, and a definitions sheet covering key concepts such as AI, data science, medical imaging, and trustworthiness. Workshop materials included a Mural board template, a SessionLab agenda, and a list of prompts for storytelling.

#### First workshop session

The first session focused on group storytelling, where participants shared personal or hypothetical experiences related to AI, ethics, and medicine. Participants were prompted using specific storytelling questions to guide the discussion. Following the storytelling, they identified ethical issues and dilemmas inherent in the stories.

To facilitate data analysis, the sessions were recorded with Microsoft Teams, with audio extracted and transcribed using the Condens tool [42]. Transcripts were anonymized, with identifying details removed, to ensure confidentiality. The anonymized transcripts are available in Ref. [43]. Facilitators also took notes on the Mural board to capture key insights.

#### Inter-session analysis

After the first session, facilitators analyzed the transcripts using MAXQDA to code key issues, dilemmas, and contextual information [44]. These were summarized on the Mural board for reference in the second session. This analysis helped prepare for the second session by thoroughly identifying the core ethical concerns raised during storytelling.

#### Second workshop session

The second session began with a review of the identified issues, dilemmas, and contexts. Participants then prioritized these topics through a structured topic selection process. Next, they engaged in a Planguage-based requirement definition phase [34], focusing on the top-voted (most critical) ethical issue. During this phase, participants defined relevant stakeholders, established scales for measuring progress, and identified parameters to quantify progress on the selected issue. At the end of the session, participants were asked to answer a post-workshop questionnaire, and facilitators recorded their impressions on a feedback sheet.

#### Post-workshop analysis

Facilitators then undertook post-workshop analysis, which included refining the defined requirements, conducting deeper content analysis of the stories shared, and incorporating the findings into the ethics framework.

### Data analysis

Content analysis was applied to the stories and comments shared by participants during the storytelling activity in two steps. The first step involved coding issues and dilemmas from the transcript of Workshop Session 1 to guide the subsequent discussion toward maximizing its value. This step involved familiarization with the data and deductive generation of initial codes. The second step, conducted after the workshops, focused on identifying an underlying structure of recurrent themes by analyzing patterns in word usage, relationships between concepts, and context [45–47]. The analysis served two objectives: (i) to uncover participant concerns about operationalizing AI ethics, and (ii) to identify ethical dilemmas, whether explicitly mentioned or implied through storytelling.

Session 1 transcripts were coded and thematized using MAXQDA 2022, [44] with codes organized into issues, dilemmas, pieces of context, and additional categories relevant to the researchers’ interests (e.g., EU HLEG requirements). Context codes were incorporated to track the situations in which specific issues were mentioned, aiding facilitators and participants in recalling when and why particular points arose. A coding standard operating procedure (SOP), provided in the Appendix, detailed the coding process to ensure consistency across the analysis.

Codes were assessed based on their frequency and whether they were mentioned by all teams. Participants were also invited to provide feedback on a near-final manuscript draft. For anonymity, each participant was assigned a unique code (e.g., G1P4 refers to Participant 4 in Group 1).

Regarding the type of content analysis, our approach was primarily manifest, as we focused on the explicit content of participant speech and workshop notes. While some minimal interpretive work was involved in grouping related issues into themes, our aim was to remain close to what was actually said during the workshops.

Concerning our epistemological stance, we now describe our approach as pragmatic, following Ref. [48]. Our goal was not to generate generalizable theory, but to explore the practical utility of a co-creation method for AI ethics operationalization.

### Trustworthiness and procedural feedback

Several practices were used to enhance the trustworthiness of the analysis. The coding process followed a clearly structured and transparent approach. Coding was primarily carried out by two researchers (LLR and VM) using MAXQDA, guided by a shared coding SOP. Sequential exchange of MAXQDA files allowed for ongoing refinement of codes and themes. This iterative process supported dependability and confirmability. To ensure auditability, snapshots of the MAXQDA file were archived at each major stage, documenting the evolution of analytical decisions.

To support reflexivity, facilitators were encouraged to keep diaries reflecting on personal assumptions and the potential influence of their role on workshop dynamics. The two researchers who were the main facilitators (LLR and CB) maintained such diaries, though their content was neither disclosed nor analyzed. While formal intercoder agreement was not calculated, the collaborative and iterative nature of the process contributed to analytical rigour.

For participant validation, individuals were invited to review a near-final manuscript or theme summary. Although their feedback was limited to expressions of agreement and did not lead to revisions, this offered a basic form of member checking. Memoing was not conducted, and while peer debriefing may have occurred informally in exchanges with co-authors, it was not systematically documented.

We completed the COREQ (Consolidated Criteria for Reporting Qualitative Research) checklist to ensure comprehensive reporting of our qualitative study. The completed checklist was included with the manuscript submission.

A procedural feedback process was implemented to assess the workshops’ effectiveness. This included: i) collecting demographic data (e.g., age, gender, ethnicity) to evaluate potential sampling and representation biases, that is, whether the participant sample reflected the broader VALIDATE consortium, and whether individual voices may have been unevenly represented due to internal hierarchies; ii) assessing participants’ satisfaction through a questionnaire; and iii) collecting facilitators’ reflections on the process. Participants rated their experiences on content quality, communication, and perceived technical and medical competence before and after the sessions. Their level of satisfaction with the diverse activities throughout the workshops was also measured. The full set of questions can be found in the procedural-feedback guide in the supplementary material. Ratings were given on a scale from 1 (strongly disagree) to 4 (strongly agree), with sentiment scores calculated to evaluate satisfaction. We computed the associated sentiment as the rating for questions toward a positive evaluation and (5 minus the rating) for questions toward a negative evaluation. Qualitative feedback was collected through open-ended questions to derive insights for improvement. The participants’ answers are available in Ref. [43]. The facilitators rated the workshops on several quality metrics, including the relevance and variety of participant contributions. Additionally, the number of stories shared in response to each prompt was monitored.

### Deviations from the study protocol

Several deviations from the original study protocol occurred. Participant recruitment numbers were lower than originally expected. The protocol estimated 40–60 participants based on a figure of approximately 70 VALIDATE project staff members. However, this number was later found to include university administrative personnel and affiliated staff not directly involved in research activities. As our target population was research staff with relevant scientific, technical, or clinical roles, the actual eligible pool was 32 people, out of which 20 were ultimately recruited for the workshops. A comparison with Z-inspection outputs was not performed, as the results were too varied in scope and structure for meaningful comparisons. Expert assessments of workshop outcomes were postponed and will be addressed in a separate interview study conducted in autumn 2024. Although concept mapping was used to guide the ethics framework, it was not applied in the content analysis of the workshop transcripts and thus fell outside the scope of this paper.

## Results

Five overarching themes emerged from the analysis: (i) priority setting of ethical issues and dilemmas; (ii) core ethical issues identified by participants; (iii) requirement formalization; (iv) issues not addressed by existing guidelines; and (v) procedural insights from the workshops.

### Overview of participants

The workshop sessions, conducted in January and February 2023, included 20 participants divided into five workshop teams (WSTs): four participants in WST1, three in WST2, three in WST3, five in WST4, and five in WST5. Participants had diverse backgrounds as detailed in Table 1.

**Table 1:** Team composition and participant professional backgrounds

WST1	WST2	WST3	WST4	WST5
Neurologist [senior] Tech/AI [senior] Admin [senior] Tech/Dev. [junior]	Neurologist [senior] Tech/AI [senior] Patient Rep. [senior]	Tech/AI [junior] Patient rep [senior] UX&Design [senior]	Tech/Engr. [junior] Tech/AI [junior] Radiologist [senior] Neurologist [senior] UX&Design [senior]	Neurologist [senior] Tech/AI [junior] Tech/AI [senior] Tech/Dev. [junior] Tech/Dev. [junior]

Participants had a rather balanced gender distribution and were predominantly aged 31–50 years and of white ethnicity. The demographic questionnaire allowed participants to identify as trans or non-binary, but all identified as cisgender. Ethnicity options in the questionnaire included: Arab or Middle-Eastern, East-Asian, South-Asian, Black or African, Indigenous, Multi-ethnic, White, and “None of the above, please specify” (Table 2).

**Table 2:** Demographics of participants

Category	Details
Age	4 participants aged ≤ 30, 16 participants aged 31–50, 2 participants aged ≥ 51
Gender	11 men, 9 women
Ethnicity	17 participants identified as white, 2 as of South-Asian descent, 1 selected “none of the above” without further specification

### Theme 1: priority setting of ethical issues and dilemmas

Ethical issues were classified in accordance with the 7 principles of the EU HLEG document, which were considered as subthemes of theme 2. Concerns in theme 4 were also classified as subthemes in an analogous way. The ethical issues that were mentioned by *all* WSTs during the first workshop session were *explainability*, *model validity, privacy*, and *model robustness*. The most frequently mentioned issues were *explainability* and *epistemic authority* (16 mentions each), followed by *fairness* (12 mentions), *model validity* (11 mentions), *transparency* (11 mentions), *privacy* (9 mentions), and *human autonomy and oversight* (8 mentions).

The ranking of the top 10 issues across WSTs revealed diverse priorities. *Validity* and *explainability* were consistently top-ranked by most teams. *Privacy* and *transparency* were prioritized highest by one team each but ranked lower by others. *Fairness* was mentioned by all groups, but was frequently ranked 5th or lower. The detailed results are provided in Table S1 in the appendix.

Common ethical dilemmas included choosing between a *global or subpopulation model* in ML design, optimizing *sensitivity versus specificity* in classification and decision making, and balancing *convenience versus privacy*, each mentioned four times. Another dilemma, the trade-off between *utility and privacy,* was mentioned three times.

### Theme 2: core ethical issues identified by participants

The analysis revealed a number of recurrent ethical concerns across workshop discussions regarding the use of AI in stroke care. Below we detail the most salient ones.

*Explainability* discussions focused on using explainable AI algorithms to understand "why the AI algorithm took that particular decision" [G4P2] and “why did the doctor also believe in what the tool told [them]” [G5P4]; to “identify feature importance” [G5P4], which might be beneficial "for doctors’ acceptance, […] sanity checks, [and] for the patients and/or families to get some trust… why the decision is as it is" [G4P1]. "Lacking explainability leads to lacking […] values, understanding and agency" [G2P2]. One clinician shared a story where an ML model to detect cerebral palsy (CP) in infants could “find cases that doctors don't find. Which is awesome, but […] we can't explain it, so we don't use it […] to protect against misuse, and also because we wouldn't get it through the medical device regulatory. But not using it means that we're missing cases of CP, which I think is unethical” [G2P2].

*Privacy* concerns included limiting data collection to the minimum required [G5P2] and potential tensions between data diversity and privacy protection: making “sure that the data sources are more diverse […] clashes with the current General Data Protection Regulation (GDPR)”, the EU’s data privacy framework [G5P2]. Participants highlighted risks of data misuse if sensitive information is not secure: “you’re tracking a lot of very sensitive data […] could be used by someone else” [G3P3]. They also referred to the challenge of preventing patient re-identification with inherently non-anonymizable data: “imaging data can never be anonymized because it’s always […] unique to that person” [G5P1]. The dilemma between privacy and convenience was mentioned in the context of social networking: “I’m trading off convenience for […] my own privacy” [G5P5].

*Model robustness* was discussed in terms of maintaining accuracy in real-world scenarios, especially when deployed in populations not fully represented in the training data or when accuracy varies across patient subgroups: “the tool has a low accuracy for some patients; then I guess it’s not reliable” [G2P2]. The challenge of maintaining model performance over time because “the demographics of the patients coming into the hospital […] shift because of [socio]economic factors” [G5P5], an instance of concept drift, causes accuracy to decline. Post-deployment monitoring systems can help detect such shifts, “you should have a system to tell you when […] you should retrain” [G5P5], which is not straightforward in regulated environments: “it might require your time to go back through all the medical device regulations (MDR) work again,” indicating a tension between model adaptability and compliance requirements.

*Epistemic authority,* the degree to which clinicians trust and defer to AI recommendations based on their perceived expertise and reliability [49], was discussed in cases where clinician and AI opinions diverged, such as when one participant stated, “the recommendation from the tool […] was against my feeling about the patient. So I decided against it” [G2P1], potentially affecting patient outcomes. Conflicts where a clinician disregards AI advice and the outcome is poor also raise liability concerns. Participants emphasized that trust in AI depends not only on the system itself but also on who is using it. As one put it, “It’s less about the characteristics of the AI system […] and more about the characteristics of the person using it” [G5P5]. Inexperience was seen as a justification for AI use: “the system would reduce the error somebody who's not experienced is making” [G5P1], but when senior clinicians were available, their judgment was preferred: “I’d rather ring [the senior doctor] and wake him up […] I’m trading off [their] sleep for more experience in my treatment.” [G5P5].

*Fairness* was discussed in relation to training models on diverse populations, socioeconomic disparities, accessibility, and systemic bias. One participant noted that “patients who are living in villages […] would usually come when the symptoms are much worse,” risking bias if models are trained mostly on urban cases [G4P2]. Accessibility concerns also included economic barriers: “only wealthy people […] get access to the AI system,” meaning care could “be based on the person’s ability to pay” [G5P5]. Others noted financial incentives may distort care: “the hospital profits if you make this thrombectomy” [G5P1], and “smaller hospitals get […] downgraded […] because they don’t have […] profitable treatments” [G1P4]. Finally, participants stressed that “historic biases” in training data could harm some groups, and that “you want to be sure that as an individual you get the best outcomes” [G4P3].

*Transparency* was discussed with a focus on system limitations, “in which cases the system can be used” [G3P1], the degree of (un)certainty [G5P4], informing about "what data has been used", and clarifying the meaning of model performance claims [G1P2]. One participant noted the importance of deployment clarity over "the characteristics of this system itself" [G5P5]. However, when designing usable and intuitive software, "you want to give [users] options that you know are the best for them or the easiest for them to use” [G1P4], designers risk omitting important information.

*Human autonomy and oversight* were mentioned in relation to informing patient decisions, the challenge for patients who cannot communicate, and the need to have a human in the loop: “I don't think any […] country would agree that the AI has the last word” [G5P1]. In this context, the concern of *deskilling,* the loss of independent decision-making skills due to reliance on AI tools, was often noted, particularly for doctors who were less experienced or working in stressful environments: “If I don't have experience with stroke patients, they come in, and I just feed all the data into the AI, and I never try to learn myself” [G5P1].

### Theme 3: from issues to requirements

In the second workshop session, participants worked to translate their selected priority issues into concrete requirements using Planguage. WSTs 1–4 translated their top-ranked issue into initial drafts of low-level requirements using Planguage, while WST5 produced fragments of a quantified requirement. Below, we present a comparison of the workshop-generated drafts and the final requirements incorporated into the ethics framework. All workshop outcomes contributed to the development of the ethical framework. A comparison between the workshop-derived requirements and the final requirements in the framework is available in Table 3.

**Table 3:** Requirements drafted during workshops and included in the ethics framework

WST	Top-ranked issue	Requirements drafted during the workshop	Requirements included in the ethics framework
1	*Privacy*	"The system complies with [relevant regulations]" "All [development materials] are stored according to [practices for privacy]" "[Data type] is available for access to [stakeholder type] through [process]"	The AI tool complies with relevant [privacy regulations] by [date] All [development materials] are stored via [practices for privacy], with practice enabled before [date] Privacy information to answer [local ethics committee questions] to approve data collection for prospective study are needed [time point] in the research data management plan
2	*Explainability*	"Explanations and [information media] relevant for stakeholders to read or understand are communicated in lay language so that it enables any explainee to protect their interests." "Explanations need to be communicated and defined in line with the [constraints] of the project" "[Relevant information] need to be translated by a science communicator when relevant and explained to [stakeholders]"	% of [explainability methods] applied in the tool are validated by [metric] by [time point] Explanations need to be communicated and defined in line with the [constraints] of the project by [time point] The system is not a black box achieved by [time point]
3	*Transparency*	"[Limitations and metadata] are available to doctors in the app during emergencies " "Tool should follow [relevant ISO norms] that are within our scope and resource limitations to prepare for compliance with the MDR during the project period." "A thorough risk assessment following the MDR should be done to check and prove which risks are involved and whether they are low enough for this tool." "The tool needs to set an intended purpose and prove that it is able to achieve this intended purpose without a certain level of risk to the patient. "	[Limitations and metadata] are available to doctors in the app during emergencies [time point] [Traceability information] is available to doctors in the app during emergencies at the end of the project [Information] relevant for [stakeholders] to read or understand are communicated in adequate language so that it enables [stakeholders] to protect their own interests and is available [time point] [Relevant information] for [explainee groups] need to be explained to [explainee groups] and translated by a science communicator when relevant Explanations are specifically tailored to [different explainee groups] [time point] The tool needs to define the intended purpose [time point]
4	*Model validity*	"X patients out of N patients whose doctors use the tool have a significantly improved mRS 90 compared to the control group" "% of [regulation requirements] are addressed to prepare for a randomized clinical trial with [n patients] that validates that mRS 90 patient outcome is significantly better when using the tool compared to current standards of care" "% of [explainability methods] are within the [measurement thresholds] or validated by clinicians"(*) "X number of relevant methods for measuring model uncertainty are used to provide a confidence score for predictions (for doctors)"(*) "% of minority [subgroups] have accuracy error rates similar to the majority for the predictions"(*) (*): Requirements more aligned with principles different from "validity"	Validity will be measured by doing a randomized clinical trial (RCT) to examine whether at least [ratio] of [RCT study size] patients whose doctors use the tool have a significantly improved mRS 90 compared to control group at [time point] % of [RCT regulation requirements] are addressed to prepare for an RCT [time point] that validates that mRS 90 patient outcome is significantly better when using the tool compared to current standards of care
5	*Validity*	"The tool has been tested and validated by randomized clinical trials that demonstrates that [inexperienced doctors] who use the tool will have an equal or better patient outcomes compared to [stroke specialists]" "The tool is tested/validated in RCTs for different [contexts/niches]"	

WST1 identified 3 privacy-related requirements: compliance with *privacy* regulations, proper storage of development materials, and processes for making data accessible to stakeholders. The ethical framework included 5 *privacy* requirements, including the approval of data collection for prospective studies. WST2 elicited 3 requirements on *explainability* and making explanations understandable for stakeholders. The framework contained 3 different requirements, with a more elaborate set of parameters, as a subsection of *transparency*, which included validating explanation methods with clear metrics. WST3 defined 4 *transparency*-related requirements, addressing system limitations, metadata availability, MDR compliance, risk assessment, and intended purpose. The ethics framework contained 4 parallel requirements, including traceability and adequacy of explanations.

WST4 and WST5 generated 7 *validity* requirements, though 3 of them were more aligned with *explainability*, *transparency*, and *fairness* (noted in italics in the table)*.* The framework included 2 *validity-*related requirements, and there was no section explicitly titled *Validity* (the challenge of working with the *validity* concept is elaborated in the next section)*.* WST4 emphasized *validity* involving success in RCTs, clinician-validated explanation methods, measurement of model uncertainty, and ensuring equitable accuracy across population subgroups. WST5 contributed *validity* requirements related to RCTs, inexperienced doctors, and varying contexts, which were included in the ethics framework under *Accuracy, Human Agency,* and *Avoidance of unfair bias* sections*.* All requirements incorporated into the framework were assigned specific time points.

### Theme 4: issues beyond the EU guidelines

The mapping analysis concluded that 6 issues raised in the first session across the workshops could not be mapped to the EU Trustworthy AI Guidelines [7]: *time sensitivity*, a concept relevant to diverse processes; *validity*, a recognized term that was used with a wide range of meanings; *prevention of harm to patients,* a common risk that may be exacerbated by the use of AI; *patient-inclusive care,* addressing the diversity of patient needs*; quality of life* and its prioritization over survival rate when possible; and the *lawsuit prevention* arising from unfavorable outcomes.

*Time sensitivity* and its variability were discussed across all workshops. Participants noted potential efficiency gains from AI in patient prioritization and time management, while ensuring fairness. However, concerns were raised about delays caused by inputting data into an AI system. One example involved a patient-traceability bracelet prototype, discontinued due to its time-consuming activation routine [G2P1]. Additionally, overreliance on AI systems designed to save time could lead to a loss of human autonomy. This issue has been identified in recent literature, [50] highlighting its potential tension with explainability.

The term *validity* was also referenced across all workshops, where it was used as a broad catch-all term addressing various desirable aspects of AI system evaluation. Participants did not always specify what type of validity they referred to (data validity, model validity, etc.). Discussions touched upon the system’s effectiveness for the relevant population, the adequacy of metrics such as the modified Rankin scale (mRS) for validation, cost‒benefit considerations, strategic decisions on sensitivity versus specificity, transparent evidence of system performance relative to current standards of care, and compliance with RCTs.

*Model validity* was defined by WST2 as "easily understandable [and accessible] evidence that this tool actually works and is useful" [G2P3], validated through randomized control trials (RCTs) and demonstration in “a large cohort of people or in [several] studies” [G3P1]. Participants noted the challenge of ensuring validity when AI systems are updated, requiring "revalidation […] in light of medical device regulations (MDR)" [G5P5].

Medicine-specific requirements include *prevention of harm to patients*: “not just treatment decisions, but [also] transfer decisions […] could come to harm because of the AI system deciding something that's not correct” [G5P4]. Other risks of harm include overdiagnosis: “a cancer that is slow growing and won’t kill the [old] person […] is not particularly useful information […] they still have the distress of knowing they have this, but there's no treatment for it” [G5P5].

*Patient-inclusive care* and *quality of life* were discussed in the context of empowering patients to express their concerns if an AI-recommended treatment chosen by doctors contradicted their values. Patients may have different preferences regarding treatment choices or the trade-off between recovery probability and quality of life improvements after treatment due to, for instance, side effects. However, such involvement can be hindered by time constraints or inability of the patient to communicate [G2P3]. The emerging challenge of ensuring patient voice representation in AI recommendations is not fully captured under generic human autonomy principles. Patient inclusiveness is reflected in medicine-specific guidelines (see, e.g., Ref. [51]).

The potential for lawsuits due to unsatisfactory patient outcomes led to the inclusion of *lawsuit prevention* as a requirement. One participant noted that a well-designed AI system might lead to “more lawsuits, but better outcomes of the lawsuits" [G1P2] by ensuring that medical decisions are based on more comprehensive evidence.

### Theme 5: workshop experience and procedural feedback

We assessed the ratings from facilitators and participants, the monitoring of storytelling prompt usage, and feedback collected from open-ended questions.

The facilitators rated the quality of the workshop outcomes on a scale from 1 (worst) to 4 (best). All WSTs received median ratings of 3 or 4. Detailed rating results are provided in Table S3 in the appendix. The main facilitator also rated the variety and relevance of participant input. Variety was rated 2 (low) for several WSTs, whereas relevance was rated 3 or 4 (high) for most WSTs. Full scores are available in Table S4 in the appendix. Moreover, in WST5, facilitators observed that the internal hierarchy within their organization made subordinates quieter than their leader.

**Fig. 2:**
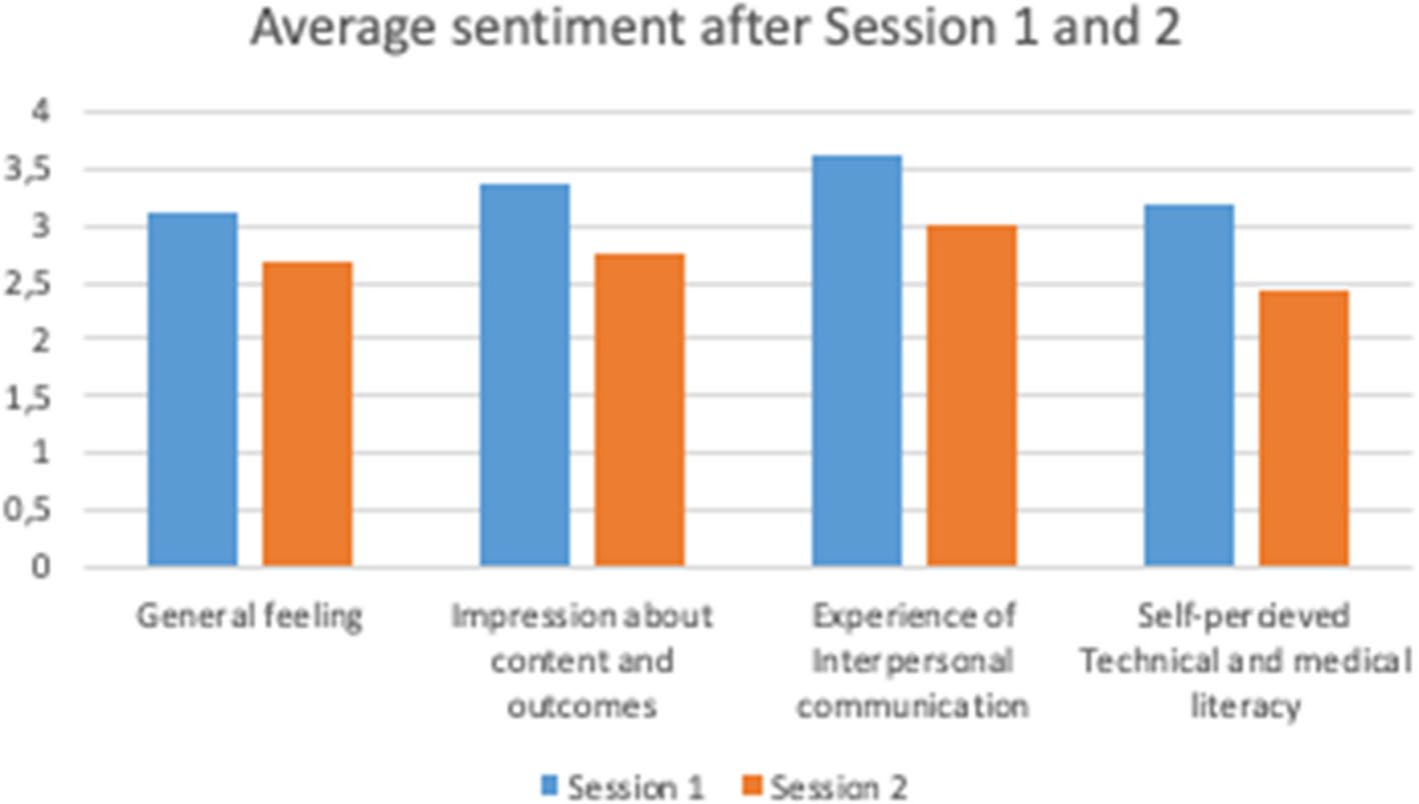
Comparison of sentiment metrics after workshop sessions

Participants completed post-workshop questionnaires covering general feelings, impressions of content and outcomes, interpersonal communication, and self-perceived technical and medical literacy. Responses were generally favorable (median between 3 and 4 on a 1–4 scale, where 4 is complete satisfaction and 1 is complete dissatisfaction), except for self-perceived technical and medical literacy, where two teams reported lower scores (median 1 or 2). Average sentiment for each domain is shown in Fig. 2. After session 1 (storytelling and issue identification), ratings exceeded 3 in all domains: general feeling (3.10), content and outcomes (3.36), interpersonal communication (3.61), and self-perceived technical and medical literacy (3.19). In contrast, Session 2 (prioritization and Planguage definition) received lower scores, none exceeding 3: general feeling (2.70), content and outcomes (2.77), interpersonal communication (3.00), and self-perceived technical and medical literacy (2.42). A histogram analysis (Figure S1 in the Appendix) indicates good satisfaction levels for Session 1, while Session 2 had more dissatisfied participants, reflecting challenges with Planguage application.

The number of responses to each storytelling prompt was tracked. Most attention was given to ethical issues in system implementation (11 responses) and technological tools conflicting with a person’s values (9 responses). For a detailed summary, see Table S2 in the appendix. Key insights from the free-text responses included concerns about the need for multidisciplinary teams versus more homogeneous groups.

## Discussion

The present study introduces a methodology for operationalizing AI ethics through co-creation workshops. Our analysis demonstrates that such a methodology effectively elicits key ethical requirements based on project staff’s perspectives in a Horizon Europe-funded medical AI project, contributing to the development of a project-specific ethical framework. It also identifies ethical issues not covered by the EU HLEG principles. The participants identified relevant issues and dilemmas, and formulated useful first drafts of quantifiable, low-level requirements to be further developed by experienced AI ethics facilitators as part of the co-creation. Overall, all WSTs identified and ranked the issues implied by their stories and succeeded in using the Planguage method to co-create low-level requirements.

Our efforts in developing and testing this workshop methodology tackle the critical task of AI ethics operationalization, which involves translating high-level ethical principles into practical, actionable requirements [12]. Despite the abundance of guidelines discussing high-level principles, there is a lack of discussion on the practical implementation of such principles [6, 12]. This is a problem since vague high-level principles fail to solve pressing normative challenges during development [13]: intra-principle normative tensions arise when we want to translate a single principle into a technological artifact, whereas inter-principle normative tensions emerge when competing ethical demands occur during AI design and development [52]. These are two main hurdles to operationalization [52]. Our methodology addresses this research gap by motivating project staff to overcome these hurdles during AI development [16], thereby addressing the nuanced ethical dilemmas encountered [13].

All WSTs provided useful content for the quantification of low-level requirements. While 4 out of 5 WSTs provided full quantified requirements, one WST provided only fragments that were, however, still useful in the following uptake (see Table 3). One possible reason for this difference is that WST5 had more junior participants than senior participants. Moreover, it was the only one comprising seniors and juniors from the same institution. The lead facilitator reported that subordinates were quieter than their leader, probably because of the internal hierarchy within their organization. To mitigate this risk, future work could implement more activities where participants individually reflect on their own before sharing with everyone in turn, as opposed to open discussion.

Overall, we concluded that the results were positive based on: i) both RQs were answered in a way that we interpret positively, i.e. a) the workshops generated rich and relevant ethical requirements for the project´s ethical framework and b) some requirement extended the scope of the HLEG Trustworthy AI guidelines; ii) the methodology proved resource-efficient; iii) the ratings reported in the procedural feedback phase were favorable. The participants experienced the workshops positively, with some challenges in formalizing requirements in a structured way (Planguage). The WSTs identified a highly diverse set of ethical issues and dilemmas, which was extremely useful in drafting ethical requirements for the Horizon Europe project by the facilitators. In the following, we address specific advantages, challenges, and limitations of the methodology based on our experience with this study.

### Advantages and challenges

A key advantage of our approach over previous methods is that the few major and relevant existing methods for AI ethics operationalization are lengthy and time-consuming. Embedded ethics, value-sensitive design, the digital catapult methodology, or Z-inspection [17, 18, 20, 53], and others demand many resources and are thus particularly challenging to scale. In contrast, our proposed method is both cost-effective and time-efficient. As evidenced by our results, only a few AI and ethics experts (potentially even only one) are needed to act as facilitators instead of involving many specialists in AI ethics. Each workshop participant was involved for only four hours, and each facilitator was involved for 20 h during workshop sessions. The facilitator responsible for coding issues, dilemmas, and associated context had to invest an additional six hours in analyzing the content of each workshop. However, this additional work was performed for research purposes and can be omitted, if necessary, when the methodology is used in practice. The workshops were designed to test the method and keep the workload for the participants small. Thus, only one out of ten issues was quantified by participants in each workshop, and the study did not reach data saturation. When applied in practice, this needs to be considered, as it may lead to longer durations for the second session.

Given the resource efficiency and scalability of our method, we do not see it as naturally opposed to established methods. Since the methodology is independent of the applied context, it can be used by independent facilitators (e.g., consultants) and hired ethicists (e.g., in the form of embedded ethics) [45]. Additionally, workshops could even be integrated into a larger assessment (such as a Z-inspection, Value-sensitive Design, or embedded ethics) as a complementary tool to foster early multi-stakeholder dialogue and engagement, when the AI system is still in the design phase.

From a practical standpoint, our workshop methodology has proven useful for bridging the gap between research and practice, leading to the identification of ethical requirements for creating an ethics framework. We evaluated the content as useful given the relevance and specificity of the requirements included in the project’s ethics framework. We also find that the EU HLEG Trustworthy AI principles are broad enough that most of the requirements elicited during the workshops and the subsequent framework generation phase can be mapped to the seven categories therein.

Results from the procedural feedback phase showed that participants rated issue prioritization and requirement elicitation in Planguage negatively, citing difficulties due to their limited technical background. The facilitator's experience further highlighted the need to improve Planguage use in this context. Our interpretation is that Planguage was too complicated in the context of a workshop with diverse stakeholders, and needs modifications in this context. One approach to addressing this issue is to allocate more time to these activities while maintaining the resource efficiency of the method. Another is to improve the efficiency of requirement elicitation by either employing facilitators experienced in Planguage, who can ask precise questions, or by providing participants with prior information on Planguage, including examples from past workshops. The latter approach is preferable, as facilitators with specific expertise may not always be available. Additionally, ensuring that participants have a solid understanding of Planguage enhances the co-creative process by allowing them greater autonomy in defining the metrics used to assess the ethical aspects of their work. While Planguage offers rigor and traceability for defining quantifiable requirements, we acknowledge that other tools (e.g., ALTAI, Moral IT Canvas, or the Ethical matrix) may offer more accessible formats for participants unfamiliar with formal planning languages.

Future projects can benefit from this research. First, researchers and/or companies can replicate and adapt the proposed methodology to suit their specific needs. Second, the workshops can raise ethical awareness among project members, encouraging them to reflect on the ethical challenges in their work. The Planguage tool can also help project managers to better control the time invested in ensuring ethical implementation. Future adaptations of our method may consider offering a choice of tools depending on the participants’ technical background and project needs.

### Methodological considerations and limitations

Despite its advantages, co-creation workshops can be influenced by participant hierarchy. Ideas from people perceived as more senior or powerful, such as doctors versus patients or professors versus postdoctoral researchers, may be more likely to be endorsed [54]. While we attempted to mitigate this when forming teams, we cannot fully rule out this effect in our workshops. Future work could implement more activities where participants individually reflect on their own before sharing with everyone in turn, as opposed to open discussion.

Allowing free discussion during storytelling leverages participants' expertise but may lead to an uneven focus on relevant themes. For instance, participants may focus on topics within their area of expertise, leaving certain issues unaddressed. Future research or adaptations of this method could guide the discussion using a predefined list of principles or requirements right after a brief (e.g., 45-min) storytelling session; as a modification to the "brainstorming" phase in comparison to our methodology.

The majority (72.70%) of the participants were between 31 and 50 years old, potentially introducing a bias against older populations or those under 30, such as younger stroke patients. For instance, a developer team in a similar context might not fully consider the limitations of individuals over 50 or those without a computer science background. Additionally, the predominance of white participants (81.80%) introduces another potential bias into workshop outcomes. In general, all data science practices must critically examine and address power dynamics to avoid perpetuating inequalities [55]. This extends to the composition of teams involved in co-creating ethical requirements, which should reflect not only ethnic diversity but also other relevant demographic and professional dimensions. A lack of such diversity may result in ethical concerns and requirements that do not adequately represent the experiences and needs of broader populations. In this context, we assume that this case is likely to be reproduced in other Horizon Europe projects, raising the question of whether a systematic solution is necessary. One could, for example, envisage a pool of diverse experts funded by the European Commission to participate in workshops and identify ethical issues in Horizon Europe projects.

The VALIDATE consortium lacked direct access to patients, with the closest representation being a member of a patient representative organization. While this is beneficial, it still falls short of adequate patient representation, which could bring more information on the lived experiences of AI's impact on patients. This can be pointed out as a blind spot of the research consortium. In fact, the patient representative emphasized the need for greater patient involvement to strengthen the project's ethical dimensions. However, direct patient stakeholder engagement (PSE) was not possible due to bureaucratic barriers around availability and payment. To overcome this in future projects, PSE should be integrated from the outset of any project, in our case in the grant writing process, with dedicated tasks for patient involvement from the start. We recommend including PSE requirements in Horizon Europe grant agreements and similar research initiatives outside the EU.

The teams in this study were formed to encourage interdisciplinary discussion, following general guidelines for diversity. Due to the small number of participants, most groups had only one clinician, and one group lacked a clinician entirely. This absence resulted in a lack of relevant stories for the group to work with. One project member with AI ethics experience suggested that involving such a clinician member and considering more homogeneous groups with varied professional profiles could improve the process. If each group consisted of participants with similar backgrounds (e.g., doctors or developers), they could potentially elicit more relevant stories and create stronger, low-level requirements. Comparing our current methodology (heterogeneous groups) with alternative approaches (homogeneous groups) could be an interesting avenue for future research.

While this study demonstrates the effectiveness of co-creation workshops as a snapshot assessment, further evaluation is needed in contexts where they are combined with more resource-intensive assessment methods such as Z-inspection or Embedded Ethics.

## Conclusion

This study presents a novel methodology for operationalizing AI ethics through co-creation workshops, effectively eliciting actionable, low-level requirements for a healthcare-focused AI ethics framework. By engaging diverse stakeholders (clinicians, AI experts, and software developers) early in the project, the approach facilitated the identification of ethical issues and the development of quantifiable requirements. Key advantages included its efficiency and the use of the standardized planning language, Planguage. However, challenges emerged related to workshop composition, the learning curve for Planguage, and limited depth of discussions due to time constraints. Future refinements will aim to enhance participant preparation, provide better support, explore applications across different AI domains and industries, and address limitations such as potential biases and the need for more in-depth discussions.

## Supplementary Information

Supplementary Material 1

## Data Availability

The datasets generated and analyzed during the current study are available in the Zenodo repository: https://doi.org/10.5281/zenodo.14334290.

## Abbreviations

AI: Artificial intelligence
CDSS: Clinical decision support system
EU: European Union
GDPR: General data protection regulation
HLEG: High-level expert group
IT: Information technology
ML: Machine learning
mRS: Modified Rankin scale
PSE: Patient stakeholder engagement
Planguage: Planning language
RCT: Randomized controlled trial
RQ: Research question
SOP: Standard operating procedure
TRL: Technology readiness level
WST: Workshop team
