# Supplementary material for: Operationalizing AI ethics in medicine—a co-creation workshop study
Source: BMC Med Ethics. 2025 Oct 29;26:150. doi: 10.1186/s12910-025-01317-y (PMC12573952; doi:10.1186/s12910-025-01317-y)
Supplement: Supplementary file 1 — Supplementary Material 1 [file 12910_2025_1317_MOESM1_ESM.docx]

Table S1 details the ranking of all mentioned ethical issues on a per WS team basis.

**Table S1: Ranking of the identified issues per workshop team**

|  | **WS TEAM 1** | **WS TEAM 2** | **WS TEAM 3** | **WS TEAM 4** | **WS TEAM 5** |
| --- | --- | --- | --- | --- | --- |
| *#1* | Privacy | Explainability | Transparency | Validity | Validity and cost‒benefit ratio |
| *#2* | Fairness | Reliability and robustness (many subpopulations) | Validity | Explainability | Accuracy |
| *#3* | Validity for relevant population | Patient-centered and inclusive care | Usability | Human oversight | Medical device validation |
| *#4* | Usability | Measuring QoL | Explainability | Robustness | Robustness (disclosing whether tool is relevant to case) |
| *#5* | Model robustness | Epistemic authority | Fairness | Fairness in data | Explainability |
| *#6* | Transparency | Human oversight | Robustness | Safety (disclosing edge cases) | Validity (optimization criterion) |
| *#7* | Measuring QoL | Data privacy/security | Epistemic authority | Automation bias | Usability |
| *#8* | Auditability | Preserving human agency | Development bias | Fairness in medical attention | Uncertainty quantification |
| *#9* | Automation bias | Validity (openly available evidence that tool works) | Autonomy | Data privacy | Fairness (avoid unnecessary thrombectomies) |
| *#10* | Epistemic authority | Fairness (gender equality) |  |  | Transparency |

Table S2 shows the storytelling prompts that were used during the first part of workshop session 1 and the tracking of the number of participant inputs (stories) linked to each prompt.

Table S2: Storytelling prompts and number of associated participant inputs (placeholder: full table available at the end of this document.)

| --- Table S2 should appear here. --- |
| --- |

Table S3 shows the scores assigned by the 3 facilitators (raters) to the quality of the workshop outcomes. Overall, the median performance rating was positive with medians across all 5 workshop teams of 3 or 4, no 1 or 2 (on a scale between 1 and 4 where 1 is worst, 2 is below average, 3 is above average, and 4 is best). WS Team 3 consistently received ratings of 3, whereas WS Team 2 had the most favorable median score of 4. WS Team 5 exhibited the highest variability. The raters consistently rated approximately 3.

Table S3: Quality rating of workshop outcomes (1--4, 1=worst, 4=best, median score from all 3 facilitators).

|  | **WS Team 1** | **WS Team 2** | **WS Team 3** | **WS Team 4** | **WS Team 5** |
| --- | --- | --- | --- | --- | --- |
| *Rater 1* | 2 | 4 | 3 | 3 | 3 |
| *Rater 2* | 3 | 4 | 3 | 3 | 2 |
| *Rater 3* | 3 | 3 | 3 | 2 | 4 |
| ***Median score*** | ***3*** | ***4*** | ***3*** | ***3*** | ***3*** |

Table S4 shows the scores assigned by the leading facilitator to the variety and relevance of the opinions and ideas shared by the participants (scoring between 1 and 4, where 1 is worst and 4 is best). During session 1, variety was rated low (2) for teams 2, 3, and 4, although their relevance was rated high (3 or 4). The teams that shared the most relevant ideas were teams 1 and 2, which scored 4 in both sessions, whereas team 4 received a low relevance score (2) during session 2.

Table S4: Variety and relevance of participants’ opinions, scored by leading facilitator.

|  |  | **WS Team 1** | **WS Team 2** | **WS Team 3** | **WS Team 4** | **WS Team 5** |
| --- | --- | --- | --- | --- | --- | --- |
| Variety of opinions/ideas | Session 1 | 3 | 2 | 2 | 2 | 4 |
|  | Session 2 | 4 | 3 | 3 | 4 | 3 |
| Relevance of opinions/ideas | Session 1 | 4 | 4 | 3 | 3 | 4 |
|  | Session 2 | 4 | 4 | 4 | 2 | 3 |

Figure S1 shows the distribution of the satisfaction levels reported by the participants during the post-workshop surveys, which specifically focused on content, including storytelling, identification of ethical issues, topic prioritization, and defining low-level requirements.


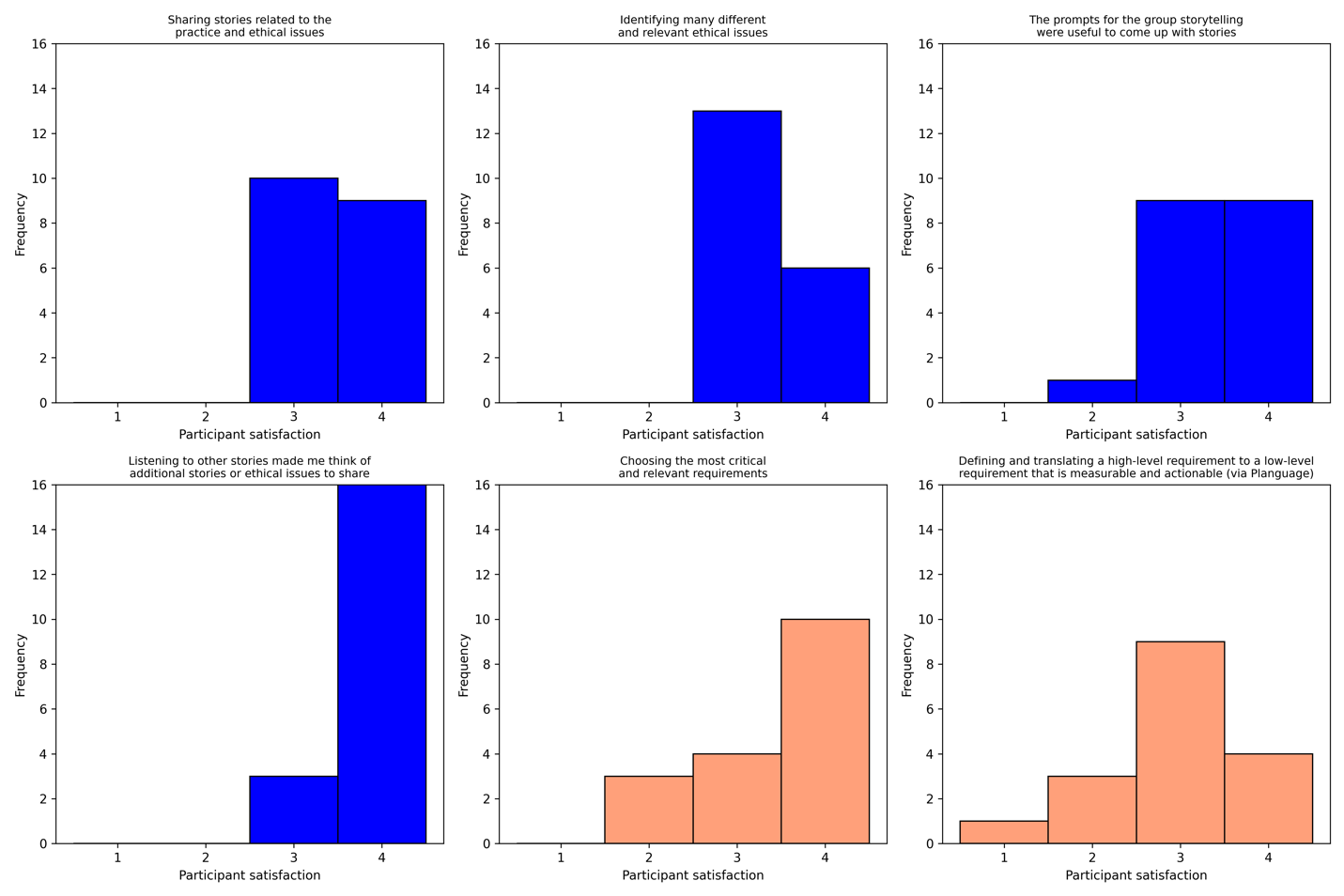


Figure S1: Histograms of the user satisfaction levels reported as answers to the content-specific questions in the survey. The blue histograms correspond to WS session 1, and the orange histograms correspond to WS session 2.

### Standard Operational Procedure: Coding WS transcripts in MaxQDA

General guidelines

Code whole sentences and not only keywords. Try for each code to cover the minimal amount of information necessary to identify the given issue/dilemma/context (see “code types” below). A transcript can be divided into conversations, and for each issue/dilemma, there should be only one code. In other words, if a participant raises an issue, then someone says something else, and if they return to the issue in the same context, there should not be two codes for each mention of the issue.

Code types

We will code sections of the transcripts from session 1 of each workshop using 3 main types of codes:

**Issues**: concerns or potential problems that may arise from the application of AI in a medical context, where there may be competing moral considerations, requiring an attentive analysis, discussion, and/or decision making.

**Dilemmas**: situations where conflicting moral values or obligations make it difficult to determine the right course or action. In this work, we code dilemmas where there are two choices, each of which has moral pros and cons, and it is not clear which one is the most ethical in each situation.

**Context**: description of situations where ethical issues or dilemmas may arise. This code is used to mark parts of the stories or comments by participants that are related to moral concerns but cannot be explicitly linked to a given issue or dilemma.

For other purposes, we use ad hoc codes:

[Ad hoc codes]: codes that are used to mark mentions of a specific topic that are not related to the above 3 categories. For example, relevant mentions of a given keyword for posterior analysis.

Auxiliary codes (to quickly identify parts of the transcript):

WS-intro rounds: Introduction rounds at the beginning of each workshop where participants talk slightly about themselves.

WS-warmup: Creativity warm-up exercises at the beginning of each session

WS logistics: Parts of the transcript where facilitators discuss how a tool works or coordinates a break or the timing of the next session.

Anonymization: Words that might be used to identify who said what in the workshop (see anonymization SOP document)

Ensuring consistency of codes: to finalize the coding process, one researcher responsible for coding must read through the whole set of transcripts and check that the coding was consistent across the different coders.

# LARGE TABLES

Table S2: Storytelling prompts and number of associated participant inputs.

|  | **Workshop Team** | | | | |  |
| --- | --- | --- | --- | --- | --- | --- |
| Prompt | **1** | **2** | **3** | **4** | **5** | Total |
| Imagine a time **when using a technological tool went against one of your values**. What happened and why did that go against your values? | 1 | 1 | 1 | 2 | 4 | 9 |
| Imagine a time in acute stroke care or other medical setting when a technological tool **was able to support your work well**. What happened? What about this tool supported your work well? | 2 | 1 | 2 | 0 | 0 | 5 |
| Imagine a time when you **decided against using a technological tool** in your clinical work. Why did you decide against that? Is there an example where you decided against that because of opposing values? | 1 | 2 | 0 | 1 | 0 | 4 |
| Do you remember a time when the implementation of a **technological tool in a medical setting led to more toxic or harmful behavior** toward patients or colleagues? | 1 | 1 | 2 | 0 | 0 | 4 |
| Have you ever experienced having to **address ethical issues during development** of a technical system? Or have you ever seen **ethical issues emerge after the system was implemented** and put in use? What were those? | 3 | 1 | 1 | 1 | 5 | 11 |
| Have you heard of ethical dilemmas that developers face when creating a technical system in a medical context or other contexts? What were they? | 2 | 0 | 2 | 3 | 0 | 7 |
| Have you ever experienced or seen situations **where opposing incentives between different colleagues/stakeholders** led to worse patient treatment? What happened? | 3 | 0 | 0 | 2 | 0 | 5 |
| Have you ever experienced or seen situations where ethically questionable/gray-area shortcuts were made in relation to a technological tool or related to a medical/health/medtech setting? What happened and why was the shortcut necessary? | 1 | 0 | 0 | 1 | 0 | 2 |
| Do you know of examples, or could you imagine a situation, where **power differences between the journey stakeholders would lead to ethical issues?** Stakeholders can be clinicians, personnel, patients, family, etc. | 1 | 1 | 1 | 0 | 4 | 7 |
| Considering the stakeholder journey from stroke to treatment, do you know of examples of **edge case patients who received worse treatment or were harmed, because the process journey/routines had not considered the circumstances or characteristics** of a such edge case patient? | 0 | 1 | 0 | 1 | 0 | 2 |
